# Revisiting the “starved gut” hypothesis in inflammatory bowel disease

**DOI:** 10.1097/IN9.0000000000000016

**Published:** 2023-01-10

**Authors:** Sean P. Colgan, Ruth X. Wang, Caroline H.T. Hall, Geetha Bhagavatula, J. Scott Lee

**Affiliations:** 1 Department of Medicine and the Mucosal Inflammation Program, University of Colorado School of Medicine, Aurora, CO, USA; 2 Rocky Mountain Veterans Hospital, Aurora, CO, USA; 3 Division of Gastroenterology, Hepatology and Nutrition, Children’s Hospital Colorado, Aurora, CO, USA

**Keywords:** metabolism, microbiota, inflammation, creatine, butyrate, innate immunity colitis, epithelium

## Abstract

Active episodes of inflammatory bowel disease (IBD), which include ulcerative colitis and Crohn’s disease, coincide with profound shifts in the composition of the microbiota and host metabolic energy demand. Intestinal epithelial cells (IEC) that line the small intestine and colon serve as an initial point for contact for the microbiota and play a central role in innate immunity. In the 1980s, Roediger et al proposed the hypothesis that IBD represented a disease of diminished mucosal nutrition and energy deficiency (“starved gut”) that strongly coincided with the degree of inflammation. These studies informed the scientific community about the important contribution of microbial-derived metabolites, particularly short-chain fatty acids (SCFA) such as butyrate, to overall energy homeostasis. Decades later, it is appreciated that disease-associated shifts in the microbiota, termed dysbiosis, places inordinate demands on energy acquisition within the mucosa, particularly during active inflammation. Here, we review the topic of tissue energetics in mucosal health and disease from the original perspective of that proposed by the starved gut hypothesis.

## 1. Introduction

Inflammatory bowel disease (IBD) is a chronic inflammatory condition of the intestine affecting more than 3 million individuals in the United States ^[1]^. Both Crohn’s disease (CD) and ulcerative colitis (UC) are significantly increasing in severity and prevalence worldwide ^[2]^. IBD is incompletely understood at present, but a number of factors are thought to contribute including changes within the microbiota, environmental exposures, and a complex set of genetic elements ^[3]^. Despite these complex factors, a pattern of abnormal innate immune responses, including within the intestinal epithelium, appear to contribute substantially to disease pathogenesis ^[4]^.

Accumulating evidence suggests that microbial dysbiosis and dysfunction of the mucosal innate immune system play central roles in the pathogenesis of IBD ^[5]^. The gut microbiota consists of the constellation of microorganisms, including bacteria, fungi and viruses, in coexistence with an intact organism. Technological advancements, especially inexpensive DNA sequencing techniques, have made it possible to understand these microbial communities at a detail never previously imagined. Studies in the past 20 years have identified a precise and consistent role for microbial communities as essential for health. Multiple mucosal surfaces, including the gastrointestinal tract, the skin, and the oral cavity provide a close and intimately interaction between the host and the microbiota. While the mammalian microbiota consists of fungi, archaea, protozoa, and bacteria, bacteria vastly outnumber the other microorganisms to the extent that the prokaryotic: eukaryotic ratio is approximately 1:1 ^[6]^. Some believe that our microbial signature is as unique as our fingerprint and only now are we beginning to understand the factors that mold this microbial population. Clearly, differences in lifestyle, diet, environment, and genetics shape the composition and activity of the gut microbiota, and ultimately our health and well-being. In this short review, we will explore how the microbiota contributes to gut energy balance health and how inflammation-associated changes to the microbiota influence disease outcome.

## 2. The “starved gut” hypothesis

Ongoing inflammatory responses are associated with profound shifts in tissue metabolism that can fundamentally influence tissue and organ function ^[7–9]^. In the 1980s and 1990s, Dr. William Roediger published a compelling series of manuscripts that probed the role of short-chain fatty acid (SCFA) transport and utilization as energy sources in human IBD ^[10–16]^. These studies strongly implicated defects in SCFA processing associated with IBD that implicated a loss of energy balance (ie, “starvation”) as a driving force in the pathogenesis of IBD.

These studies were systematic. First, using suspensions of isolated human colonocytes, he examined oxygen consumption rates as a primary endpoint with added energy sources, including SCFA, glucose, and glutamine alone or in combination. Through the process of elimination, he concluded that butyrate is the major source of fuel for the colonic mucosa and accounts for more than 70% of the oxygen consumed by the colon ^[10]^. It is now understood that the colonic epithelium consistently favors oxidative metabolism of butyrate ^[17,18]^. In the colon, for example, the insatiable metabolism of butyrate depletes local O_2_ to the extent that hypoxia-inducible factor (HIF) is stabilized and transcriptional targets of HIF are activated ^[19]^. HIF target genes that regulate metabolism classically include glycolytic genes ^[20,21]^, major butyrate transporters (eg, MCT4) ^[22,23]^ and pyruvate dehydrogenase kinase (PDK) ^[24]^. The induction of PDK within the mucosa inactivates pyruvate dehydrogenase and results in a decreased utilization of glucose as a source of acetyl-CoA within the tricarboxylic acid (TCA) cycle. As a result, the β-oxidation of butyrate becomes the primary energy source of intestinal epithelial metabolism. To compound this metabolic pathway, the functioning of butyrate as an HDAC inhibitor (see later) induces PDK1 expression, thereby further diminishing glucose utilization ^[25]^. Given this unique metabolic adaptation within the mucosa, it is not surprising that more that 70% of oxygen utilized by human colonic epithelial cells occurs through butyrate β-oxidation ^[10]^. Some regional differences were also demonstrated, where the distal colon was found to be more dependent than the proximal colon for butyrate as the major energy source. Second, he suggested that changes in the SCFA reflect the degree of inflammation in UC. This study reported that concentrations of SCFA were increased in severe UC and that butyrate, in particular, was raised in all severities of disease. It was suggested that decreased absorption of SCFA accounted for the observed increase. While this latter observation has been debated (see below), these studies lead to the major hypothesis that IBD is an energy-deficient disease. He reported that colonocytes harvested from UC patients were deficient in butyrate oxidation and postulated that the disease has a substantial “starvation” component ^[16]^. In the backdrop of deficient butyrate oxidation, the harvested colonocytes manifested increased glucose and glutamine oxidation. It is also notable that mitochondria in the epithelium of patients with IBD are reduced in number, altered in subcellular location, and dysmorphic in appearance ^[26]^, thus compounding the multiple defects in overall energy procurement.

## 3. Deficiencies in butyrate metabolism and transport in IBD

### 3. 1. Butyrate transport

**Figure 1.**
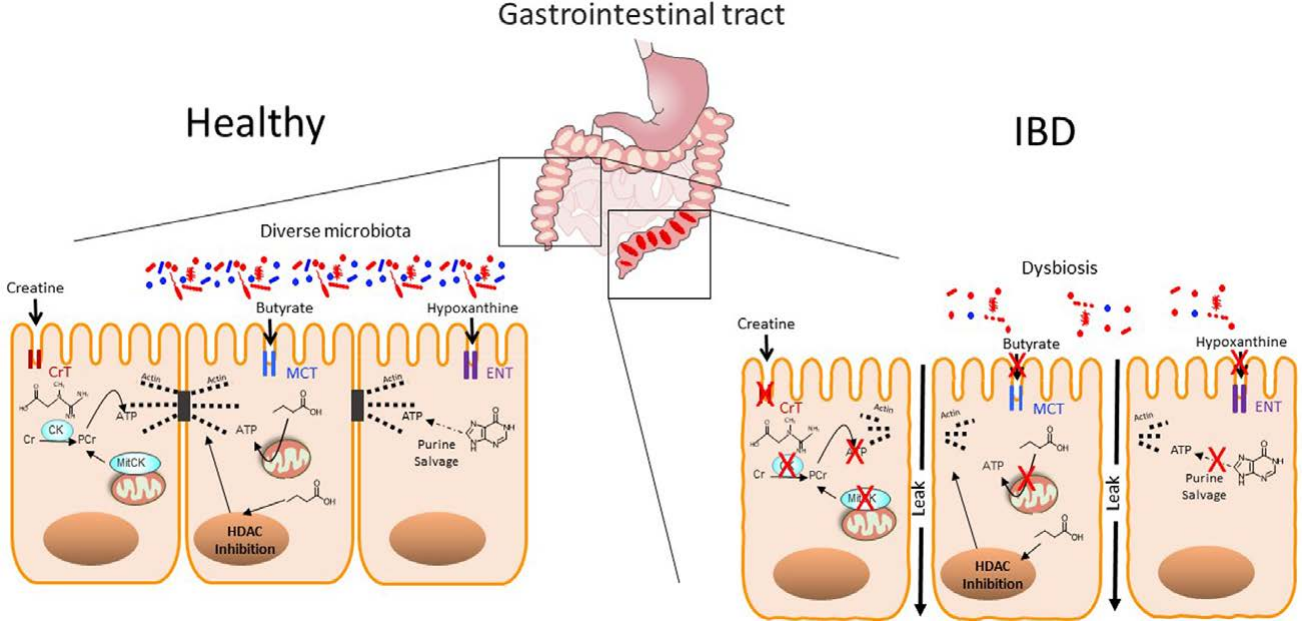
Comparison of mucosal energy procurement in healthy and IBD tissue. In the healthy mucosa (shown on left), energy within the mucosa is derived from the diet and from the microbiota. Highlighted here is the high energy demand needed to maintain the epithelial apical tight junction-actin complex. Creatine is primarily derived from the diet whereas short-chain fatty acids (esp. butyrate) and purines (esp. hypoxanthine) are derived from microbial sources and are captured as substrates for ATP generation that supports actin polymerization of G-actin to F-actin in the apical epithelial TJ complex. During bouts of active IBD (shown on right), the combination of diminished nutrient absorption and dysbiosis result in a “starved gut” phenotype that manifests in defective mucosal barrier and perpetuation of disease. See text for details. CK: creatine kinase, CrT: creatine transporter, MCT: monocarboxylic acid transporter, ENT: equilibrative nucleoside transporter, HDAC: histone deacetylase.

Consistent with Roediger’s original studies, transport of butyrate appears to be significantly diminished in UC (Figure 1). Butyrate transport occurs through at least two mechanisms, including the H^+^-coupled MCT1 and MCT4 transporters and the Na^+^-coupled transporters SMCT1 and SMCT2 ^[27]^. MCT1 is expressed on the apical and basolateral IEC membrane, whereas MCT4 exists only basolaterally. SMCT1 and SMCT2 are expressed exclusively on the apical IEC membrane. A number of studies have demonstrated that inflammatory mediators such as TNFα and IFNγ repress MCT1 and SMCT1 expression and function in IEC ^[28,29]^. A meta-analysis of 12 datasets comparing expression of MCT1, SMCT1 and SMCT2 in healthy and UC colonic tissue revealed a significant (range 17%–95%) decrease in MCT and SMCT expression in 10 of the 12 studies examined ^[27]^. Whether such decreases could account for the deficit in host butyrate is unclear, it is notable that MCT1 itself is upregulated by butyrate ^[30]^ and suggest decreased overall microbiota-derived butyrate likely contributes.

### 3. 2. HDAC inhibition

Beyond energy provision, SCFAs fundamentally regulate gene expression. Butyrate, for example, potently regulates transcription through HDAC inhibition (Figure 1). HDACs represent a family of enzymes that selectively remove acetyl groups from lysines on histones and nonhistone proteins. HDACs can either inhibit or promote transcriptional activity, depending on the individual promoter and open or closed nature of chromatin. HDACs are generally classified in four groups: class I (HDAC1, 2, 3, 8); class II (HDAC4, 5, 6, 7, 9, 10); class III (SIRT1-7); and class IV (HDAC11), originally dependent on their homology to yeast HDAC. Propionate and acetate show little to no HDAC inhibitory activity ^[31]^. Inhibiting the deacetylation of histones allows genomic DNA to remain accessible to transcription machinery. HDAC inhibition impacts expression of ~2% of mammalian genes ^[31,32]^. Butyrate also stabilizes HIF, a master transcription factor in the mucosa ^[8]^, via direct inhibition of the prolyl hydroxylase (PHD) enzymes that regulatorily degrade HIF ^[33]^ and limitation of oxygen availability necessary for PHD function through increased oxygen utilization through energy-harvesting butyrate metabolism ^[19]^.

### 3. 3. Shaping the immune response

There is significant interest in understanding how butyrate and other SCFA influence immune responses. One of the more recent mechanisms of action include SCFA-mediated activation of G-protein–coupled receptor (GPR) signaling. GPR41 or free fatty acid receptor 3 (FFAR3), GPR43 or FFAR2, GPR109A, and GPR164 have been identified as SCFA receptors, with FFAR2 and GPR109A being more specific for butyrate ^[34,35]^. Activation of these receptors on IEC trigger the production and secretion of interleukin-10 (IL-10) and IL-18 to suppress inflammation ^[36]^. Signaling through these GPRs is not limited to IECs. Regulatory T cells (Tregs) express SCFA-responsive G-protein coupled receptors (GPCRs), the ligation of which promotes histone H3 acetylation of the FoxP3 promoter to influence Treg differentiation ^[37,38]^. Furthermore, butyrate suppresses T_H_17 cells through GPCR activation to promote Treg and intestinal homeostasis in colitis ^[39,40]^.

### 3. 4. Cytoskeletal regulation

A recent genome-wide single cell RNA profiling of SCFA-regulated gene expression identified a cytoskeletal cluster of genes regulated by butyrate-elicited HDAC inhibition ^[41]^. Based initially on the regulation of barrier function and wound healing, this unbiased approach revealed a prominent induction of gene transcripts important in cell migration, tight junction formation/maintenance and myosin/kinesin-based motor complexes (Figure 1).

Pursuance of one of these butyrate targets, namely synaptopodin (SYNPO), revealed the novel expression and function of cytoskeletal regulation in the mucosa. Originally described in kidney and post-synaptic densities ^[42,43]^, SYNPO belongs to the class of proline-rich actin-associated proteins that regulate cell shape and motility ^[44–46]^. These studies with butyrate revealed that depletion of the microbiota abrogated SYNPO expression in the mouse colon and could be rescued with butyrate repletion. Model colitis studies in *Synpo*-deficient mice demonstrated exacerbated disease susceptibility and increased intestinal permeability, revealing a new role for butyrate as a mechanistic link between microbiota-derived butyrate and barrier function. As such, these studies highlight the implications of SCFA dysregulation beyond mere energy imbalance in the intestinal mucosa.

## 4. Deficiencies of creatine metabolism and transport in IBD

Beyond defects in SCFA assimilation and utilization, it is more recently appreciated that storage pools of energy may also be deficient in IBD. These storage pools, or energy reserves, are provided largely by the phosphocreatine (PCr) shuttle, and recent investigations have defined an important role for creatine in intestinal inflammation. Approximately 50% of creatine is obtained through our diet and 50% is synthesized endogenously ^[47]^. Dietary creatine is minimally digested with almost 100% absorbed in passage through the small intestine ^[47]^.

The creatine kinase (CK)/PCr shuttle, with Cr as an energy precursor, plays a critical physiological role for cells and tissues with high and fluctuating energy requirements (Figure 1). CK exists as four distinct CK isozymes, expression varies by tissue and by developmental stage. The muscle isoform of cytosolic CK (CKM) is expressed to the greatest extent within sarcomeric cardiac and skeletal muscle. The more ubiquitous cytosolic brain isoform of CK (CKB) is expressed most highly in non-muscle cells ^[48,49]^. Two mitochondrial CK (mtCK) isoforms have also been characterized, the ubiquitous mtCK and the muscle-specific sarcomeric mtCK, both of which localize to the mitochondrial intermembrane space ^[48,49]^. The CK system is essential for cellular energy homeostasis, especially in tissues with highly dynamic energy demands such as the muscle, brain, and the intestine ^[48–51]^. Within individual cells, CK facilitates the shuttling of high energy phosphates in the form of PCr between sites of ATP generation in the mitochondria and cytosolic locations of ATP consumption. As such, the diffusion limitations and thermodynamic limitations of using ADP and ATP for the storage of high energy phosphates are overcome by PCr/Cr shuttling ^[48–51]^.

Intestinal epithelial cells (IEC) predominantly express the cytosolic brain-type BB-CK and mitochondrial mtCK isoenzymes ^[52]^. Additionally, a specific creatine transporter (CrT1), belonging to the X-linked gene SLC6A8, as a member of a solute carrier family, is present in the apical cell membrane of IEC ^[53]^. Electrogenic Na^+^- and Cl^−^-dependent Cr transport, with high affinity for Cr (K_m_ ~30 µM), allows IEC to import Cr ingested with the diet (eg, from protein sources such as fish or meat) as the most significant sources for Cr ^[54]^. As CrT1 function is dependent on Na^+^, inhibition of the Na^+^/Ka^+^-ATPase (eg, through the action of Lyn kinase activation), also inhibits Cr uptake into cells, as shown recently ^[55]^.

### 4. 1. Deficiencies in CK expression

Initial insight in defects in the Cr-PCr shuttle in IBD were founded on an unbiased screen of IEC target genes regulated by the transcription factor HIF. This analysis revealed that the IEC CKs (both cytosolic BB-CK and mtCK) and the major CrT SLC6A8 are all coordinately regulated by HIF ^[56]^. Additional analysis revealed that cytosolic BB-CK enzyme localizes to apical intestinal epithelium cell adherence junctions, where it is centrally involved in ATP-dependent tight junction assembly, epithelial integrity, and barrier function. The analysis of transcripts from 30 IBD patient samples (including Crohn’s disease and ulcerative colitis) showed a significant reduction in all isoforms of CK compared to healthy patient controls (Figure 1). From this perspective, it is not surprising that IBD tissues show defects in barrier function. It is known, for example, that the tight association between epithelial tight junctions and adherens junctions to the actin cytoskeleton is a significant energy for the mucosa, where up to 25% of basal ATP is utilized to maintain barrier function ^[57]^. Therefore, the microbial dysbiosis and CK deficiencies associated with IBD likely contributes to overall energy reserves, resulting in the potential for barrier dysfunction during active disease flares.

Studies subsequent to the initial identification of deficiencies in Cr-PCr shuttle among IBD patients have been revealing from the perspective of drug response indicators and biomarker identification. First, an unbiased nuclear magnetic resonance (NMR)-based profiling of urine and serum metabolites from 43 IBD and 17 healthy control subjects revealed a prominent decrease in serum Cr among actively inflamed UC and Crohn’s disease patients ^[58]^. A more recent analysis revealed that serum Cr were significantly lower in UC patients that may be related to amino acid malabsorption ^[59]^. Second, a retrospective study of 131 IBD patients demonstrated significantly higher serum CK (undifferentiated isoform) levels in subsets of patients receiving anti-TNF therapy. High serum CK was greatest in male vs female patients and in the nonsmoker vs smoker cohort. Similar elevations in serum CK levels were noted in patients receiving anti-leukocyte adhesion molecule therapy ^[60]^. Third, a recent clinical trial evaluated the efficacy and safety of an oral selective inhibitor of Janus kinase 1 (JAK1) in UC. This phase 2b trial incorporating 250 patients revealed an increase in circulating CK levels in response to JAK1 inhibition ^[61]^. A 30-month follow-up indicated showed continued elevation of CK with chronic treatment ^[62]^. It remains unclear if CK serves as an adverse event to therapy or as a biomarker of treatment response. Finally, Starr et al recently reported that colon biopsies from pediatric IBD patients with active disease show decreased CKB expression, particularly when biopsies are harvested from areas of active disease vs areas of mucosal healing ^[63]^. It is also notable that Crohn’s patient responders to IBD-targeted therapy also expressed higher levels of CK than drug naive patients. Taken together, these observations provide some evidence that CK could serve as a biomarker of disease and may suggest drug response in some incidences.

### 4. 2. Defects in Cr transport within IBD patients

Creatine transport occurs primarily in the small intestine via the apical CrT. CrT expression has been examined in IBD patients. In one study of mucosal biopsies from 30 patients with Crohn’s disease and 27 patients with UC both showed lower expression levels of CrT1 gene transcript (Figure 1)^[64]^. Supporting evidence in cultured IEC and in colonoids from CrT1-deficient revealed that diminished Cr transport results in reduced epithelial barrier function that tracks to defects in metabolism and actin polymerization. Localization of IEC CrT1 revealed that this transporter is expressed specifically in areas of epithelial tight junctions and overexpression or targeted knockdown of CrT1 in an IEC cell line implicated intracellular creatine concentration as a driving force for epithelial barrier formation and ultimately wound healing. In IECs lacking CrT1, metabolism shifted toward a glycolysis-predominant energy metabolism that was reflected by leaky tight junctions, aberrant expression and localization of tight junction proteins and dysregulated actin polymerization similar to findings in CK-deficient IECs. In addition to the above study, a screening of 36,530 germline mutant mice for susceptibility to DSS colitis identified a mutation in glycine amidinotransferase (GATM) enzyme necessary for creatine biosynthesis. GATM-deficient colonocytes similarly showed evidence of metabolic stress and creatine supplementation was protective in GATM-deficient mice ^[65]^. These findings support the concept that energy deficiencies within the mucosa support defects in barrier formation and decreased wound healing responses.

Similar to the evidence provided above for SCFA, these studies of the Cr-PCr shuttle in human IBD argue for Cr supplementation as an adjuvant therapy to promote epithelial wound healing to promote mucosal inflammation restitution through enhanced epithelial energetics. Such a study has been suggested ^[66]^.

## 5. Purine metabolism in IBD

Most recently, it has been shown that the salvage of purine molecules can be a significant source of energy within the mucosa, particularly during stress. In models of inflammation and hypoxia, energy flux results in the pooling and salvage of hypoxanthine, which in turn becomes a major building block for the regeneration of ATP ^[57]^. This is important, because IECs have a low capacity for the de novo synthesis of purines and instead rely on well-established salvage pathways for adenylate biosynthesis ^[67–69]^, and ultimately the production of ATP (and storage forms, such as PCr). In this regard, the unique environment of the mucosa was more clearly delineated with the discovery that the intestinal microbiota is a major source of purines, termed microbiota-sourced purines (MSP) within the salvage pathways (Figure 1)^[70]^. These MSPs were discovered through analyses of water-soluble fecal metabolites in mice that identified readily measurable levels of salvageable purines, particularly in the form of hypoxanthine and xanthine. Animals lacking MSP (eg, germ-free or antibiotic-treatment) showed a propensity for increased ER stress and a loss of barrier function. Conversely, gain-of-function studies with MSP-sufficient bacterial mutants alleviated ER stress and promoted colonic barrier, thereby establishing MSP and a critical metabolic component for tissue homeostasis.

Purine salvage have also been shown to be important in human disease. For example, in a double-blind trial of 81 active UC patients with and without FMT, it was shown that the salvage of purine nucleobases (eg, hypoxanthine, xanthine, and adenine) tracked with FMT-associated disease remission ^[71]^. Patients that did not achieve remission in this trial were enriched in a microbiota that lacked SCFA production and diminished purine production, providing valuable insight into the contribution of tissue energetics to inflammatory resolution. Furthermore, purine metabolism was identified as a novel host-microbial metabolic pathway in patients with irritable bowel syndrome (IBS), a multi-factorial disease of recurrent abdominal pain and discomfort ^[72]^. Stool samples from IBS patients showed significantly lower levels of MSP, with this starvation phenotype shown to be the result of increased degradation by the microbiota and host alike, revealing a potential novel mechanism underlying IBS pathophysiology ^[73]^.

## 6. Conclusions

In his original manuscript, Roediger wrote “To unify the present metabolic findings in colonocytes with clinical features of UC and histological changes in the diseased mucosa, the term “energy-deficiency” is proposed. Low levels of free CoA and diminished oxidation of fatty acids are the basis for using the term “energy deficiency”. Usually, β-oxidation of fatty acids such as *n*-butyrate is a far more efficient source of energy supply than glycolysis, and the faulty oxidation reflects a state of “energy deficiency” ^[16]^.

In this short review, we summarize changes associated with metabolic deficiencies in active inflammation and IBD. An important question that remains unanswered is whether inflammation-associated dysbiosis is cause or effect in IBD and other related disorders. It is clear that tissue metabolism during inflammation shifts toward a less tolerant environment for strict anaerobes (eg, butyrate producers such as Firmicutes and Bacteroidetes) toward a less diverse microbiota with higher tolerance for the outgrowth of facultative anaerobes (eg, more virulent Proteobacteria) ^[74]^. While the cause of IBD is much debated, a common feature is inflammation which follows a clinical course that can be episodic or chronic. Important insight into the role of dysbiosis has been gained through longitudinal studies. For example, IBD-related dysbiosis improves with treatment but nonetheless persists even in patients that have achieved full mucosal healing ^[75]^. Such observations suggest that dysbiosis, as defined by our current understanding of disease-associated microbiota, is a fundamental feature of IBD.

It is also notable that the dysbiosis associated with active intestinal inflammation has resulted in some clinical progress. Fecal microbiota transplantation (FMT), for example, has shown great success in the treatment of active *Clostridium difficiile* colitis (CdC), where up to 90% of patients are cured with a single treatment ^[76]^. It is also notable that in a small cohort of patients, fecal samples filtered to remove bacteria show nearly equivalent efficacy ^[77]^, implicating a non-bacterial source of disease resolution (eg, microbial metabolites). Studies assessing FMT for the treatment in UC has been less promising than CdC, but generally suggest that positive FMT outcomes promote energy homeostasis associated with an enrichment of microbiota that promote SCFA production and purine salvage ^[71]^.

Taken together, even upon reflection and on the backdrop of more sophisticated technologies and advanced experimental approaches, Roediger’s original “starved gut” hypothesis appears to have survived the test of time. The presumption that energy homeostasis within the mucosa is central to the resolution of active inflammation has proven to provide an essential insight to our understanding the healthy mucosa. A more refined understanding of the composition of the microbiota and the subsequent discovery of other energy metabolites important within this conversation have resulted in previously unimaginable opportunities for the treatment of patients with IBD and likely other mucosal diseases into the future.

## Author contributions

SPC, RXW, CHTH, GB, and JSL wrote the paper with input from all authors.

## Conflicts of interest

No conflicts of interest exist for any aspect of the work presented here.

## Funding

This work was supported by the National Institutes of Health (grants DK050189, DK103712, DK104713, DK129410, and DK095491), the Crohn’s and Colitis Foundation and by the Veterans Administration (award BX002182).
